# News and Notes

**Published:** 1911-04

**Authors:** 


### NEWS AND NOTES.

### MEDICAL ASSOCIATION OF GEORGIA. ROME, GEORGIA, APRIL 19, 20, 21, 1911.

Preliminary Program.

1.—The Association of Cataracts with Uncinariasis. (Hook Worm) .................... F. Phinizy Calhoun, Atlanta

2.—Nausea and Vomiting, their Clinical Significance ........ ............................ W. W. Jarrell, Thomasville

3.—An Operative Plan for the Correction of Relapsed and Untreated cases of Club Foot ...... ..Michael Hoke, Atlanta

4.—On the Treatment of Fractures ...................... ......................... Thomas J. Charlton, Savannah

5.—Rationelle of All Treatment applied to Pulmonary Tuberculosis ..................... J. Monroe Anderson, Pinedale

6.—The Treatment of Digestive Disturbances by the Surgeon .................................. R. P. Glenn, Columbus

7.—The Treatment of Pneumonia—with Especial Reference to the Use of Alcohol ....... ... W. B Hardman, Commerce

8.—Drinking Water with Meals—a Physiologic and Dietetic Study ......................... Geo. M. Niles, Atlanta

9.—A Plea for the Systematic Examination of School Children's Eyes, Ears and Throats .... J. Lawton Hiers, Savannah

10.—Psychotherapy and Re-education in the Treatment of Nervous Diseases ................ E. T. Gibbs, Gainesville

11.—The Complete Tearing Away of the Scrotum by a Gin Belt .............................. S. B. Little, Colbert

12.—Surgical Deliberation—a Plea for Careul Consideration and Accurate Diagnosis before Operation ............. ..........................Floyd W. McRae, Atlanta

13.—Personal Experience with "606" or Salvarsan in the Treatment of Syphilis .............. Edgar G. Ballenger, Atlanta
14.—Placenta Praevia—Diagnosis and Treatment ..........
.................................. C. T. Nolan, Marietta
15.—A Clinical Study of Some Cases of Sudden Death ....
.............................. Lewis M. Gaines, Atlanta
16.—Reasons for Doubting the Corn Theory of Pellagra ....
........................... Stewart R. Roberts, Atlanta
17—Commercial Value of Sanitary Measures, Civic and Private
................................ W. A. Jackson, Atlanta
18—Salvarsan or "606" in the Treatment of Syphilis, with Report of Cases .................. W. L. Champion, Atlanta
19.—Perforating Gunshot Wounds of the Abdomen with Report of Cases ....................... J. R. B. Branch, Macon
20.—Treatment of Pulmonary Tuberculosis by Artificial Pneumothorax, Showing Author's Apparatus, S. T. Harris, Valdosta
21.—Extaueterine Pregnancy ...... A. J. Mooney, Statesboro
22.—Goitre and its Surgical Treatment with a Report of Two Cases ............................ W. P. Harbin, Rome
23.—A Hernia Case of Historic Interest, .............. ....
.............................. Henry M. Michel, Augusta
24.—The Success of a Small Sanatorium in a Small Town ..
.................................. R. C. Woodard, Adel
25.—264 Consecutive Cases of Tuberculosis among Insane Negroes ........................ N. P. Walker, Milledgeville
26.—Diseases of the Gums .............. Robin Adair, Atlanta
27.—A Plea for More Thorough Understanding and Treatment of Ophthalmia Neonatorum .. Hugh M. Lokey, Atlanta
28.—Removal of Large Pus Kidney and Large Ovarian Cyst
............................ H. R. Dondaldson, Atlanta
29.—The Diagnosis and Treatment of Intussusception ........
......................... W. Whatley Battey, Jr., Augusta
30.—Some Observations about the Alimentary Canal ......
................................ J. C. Brock, Carrollton
31—Pellagra—An Actual Case with Progress and Treatment ..
.............................. L. R. Bryson, Gainesville
32.—The Gall Bladder ................. J. L. Campbell, Atlanta

33.—A Singular Case ........... W. B. Sandifer, Blakeley
34.—Tobacco Amblyopia .............. A. W. Stirling, Atlanta
35.—Hysteria ........................ Wesley Taylor, Atlanta
36.—Stools of Children. Indications for Change of Diet and Treatment ............................ B. B. Jones, Metter
37.—Therapy of Pulmonary Tuberculosis, L. C. Rouglin, Atlanta
38—Osteosarcoma of the Pelvis with Report of Case ........ ................................. S. T. Barnett, Atlanta
39.—The Surgical Treatment of Goiter .. E. G. Jones, Atlanta
40.—Bacterial and Serum Therapy, Ralston Lattimore, Savannah
41.—The Treatment of Cancer of the Breast .............. ................................. F. K. Boland, Atlanta
42.—Potpourri ..................... E. C. Cartledge, Atlanta
43.—The Laxity of Laws Regulating the Issuance of Marriage Licenses; with Especial Reference to the Need of Medical Inspection of Parties Intending to Marry .............. ................................. W. W. Mangum, Rome
44.—Does It Pay? ................. E. J. Spratling, Atlanta
45.—Epilepsy with Report of Some Experiments in Producing Convulsions in Rabbits ............ J. N. Brawner, Atlanta
46.—Enucleation of Prostate by "Two-Step" Method ...... ................................. A. L. Fowler, Atlanta
47.—Some Surgical Difficulties Encountered by the Country Practitioner .................... Dallas Williams, Folkston
48.—The State Sanitarium for Tuberculosis ................ .......................... C. H. Richardson, Montezuma
49.—The Sinificance of Indicanuria in Surgical Cases .... .......................... W. F. Westmoreland, Atlanta
50.—Baccilli Carriers .................. K. R. Collins, Atlanta
51.—Prolonged Intubation Case ............ G. B. Smith, Rome
52.—The Power of Ignorance .............. R. P. Cox, Rome
53.—Why the Medical Association of Georgia Should Lend Its Influence in Revising the Present Committment Laws of the Insane in Georgia .................. J. C. King, Atlanta
54.—A Simple Technique in Gynecologic and Obstetric Perineal Repair Incomplete and Complete .. R. R. Kime, Atlanta

55.—The Diagnosis and Treatment of Maize Poisoning .... ............................... H. F. Harris, Atlanta
56.—Thrombosis of the Cavernous Sinus with Report of Case ............................ H. H. Martin, Savannah
57.—Typhoid Fever—Report of a Case .................. ............................. J. E. Summerfield, Atlanta
58.—The Backward Child and What To Do for It .......... ............................. Theodore Toepel, Atlanta
59.—Apomorphia and Its Uses ........ S. A. Visanska, Atlanta
60.—(Title to be announced) ............ E. B. Block, Atlanta
61.—Chronic Naso-Pharyngeal Bursitis (Thornwaldt's Disase) .................................. Dunbar Roy, Atlanta
62.—Angina Pectoris ................. J. E. Paullin, Atlanta
63.—(Title to be announced) ...... W. Z. Holliday, Atlanta
64.—Preventable Deafness .............. W. C. Lyle, Augusta

---

### NATIONAL CONFEDERATION OF STATE MEDICAL EXAMINING AND LICENSING BOARDS.

The Twenty-first Annual Convention of the National Confederation of State Medical Examining and Licensing Boards was called to order at the Congress Hotel, Chicago, Ill., by the President, Dr. Joseph C. Guernsey. Dr. George W. Webster, of Chicago, Chairman of the Committee on Arrangements, delivered a cordial address of welcome, which was ably responded to by Dr. Lee H. Smith of Buffalo.

The President delivered the annual address, choosing for his subject "Medical Licensure." The report of the Secretary-Treasurer, Dr. George H. Matson, was read, audited and approved. The report of the Committee on Clinical Instruction by Dr. Henry Beates, Chairman, and that on Materia Medica by Dr. Murray Galt Motter, were read, referred for publication and the committees continued. The report of the Committee on Mr. Flexner's paper, published in the proceedings of 1910 was read by Dr. N. P. Colwell. After an extended discussion the report was adopted as read and the committee dischared.

The symposium on "State Control of Medical Colleges" was discussed from the viewpoints of State, Law, The Medical Colleges, State Medical Examining and Licensing Boards and the Medical Profession. From the viewpoint of the State, Charles William Dabney, Ph.D., LL.D., Pres. of the University of Cincinnati, read a paper in which he contended that the State could control and conduct medical colleges more efficiently than corporations an private individuals. From the same viewpoint, Mr. Abraham Flexner, of the Carnegie Founation for the Advancement of Teaching, New York City, read a paper on "The Duty of the State in the Control of Medical Colleges," advocating this system. From the viewpoint of the Law, Hon. Chalres Alling, Jr., Chicago, read a paper giving his opinion that the courts would uphold the system. Dr. Arthur Dean Bevan, Chicago, discussed the question from the viewpoint of the Medical Colleges, setting forth the advantages of State Control (a), as regards uniformity of requirements and methods (b), as giving adequate financal support. From the same viewpoint, F. C. Waite, A. M., Ph.D., Cleveland, forcefully and hurriedly pointed out th eevils nherent under the present system and expressed the opinion that the spirit of competition and ommercialism would be eradicated if the state controlled the medical colleges. Dr. Frank Winders, Columbus, O., read a paper in which he contended that with aid rendered by the State, medical education would become more efficient by having all teachers receive a compensation commensurate with their labor, and by having a larger number devote their time to teaching than now obtains. From the viewpoint of the State Board of Medical Examiners, Dr. Edward Cranch, Erie, Pa., declared that the medical boards could more efficiently enforce the laws regulating the practice of medicine and the requirments of the board if medical education were under state control. From the same viewpoint, Dr. James A. Duncan, Toledo, presented a paper on the subject "If Medical Colleges were under State Control, would the state medical boards be enabled to determine more fully the standing?" which question 'he answered in the affirmative. For the Medical Profession Dr. Royal S. Copeland, New York City, said that if medical colleges were under state control, the medical profession would be more uniformly and efficiently educated and trained than by the present system. Dr. Horace J. Norton, Trenton, N. J., presented a paper in which he held that since the medical colleges are the source of the medical practitioner upon which devolves the care and welfare of the people, they should be under State Control. Special papers on the following subjects were presented: "The Necessity of Establishing a Rational Curriculum for the Medical Degree," by Dr. Henry Beates, Philadelphia; "Some Thoughts on the Supervision of Medical Colleges and the Conducting of State Examinations," by Dr. James A. Egan, Springfield, Ill.

The attendance was the greatest in the history of the Confederation, and the enthusiasm which began at the opening continued throughout the session. All papers were earnestly and intelligently discussed, the interest becoming so intense that it was necessary to limit the period of the discussions.

The Oregon State Board of Examiners, the Louisiana State Board of Medical Examiners (Regular), Dr. R. S. Copeland, New York City; Dr. James H. McDonald, Pittsburg; Dr. F. P. Lawrence, Columbus, and Dr. C. M. Hazen, Bon Air, Va., were admitted to membership in the Confederation.

The following officers were elected:

President, Dr. Charles A. Tuttle, New Haven, Conn.; First Vice-President, Dr. James A. Egan, Springfield, Ill.; Second Vice-President, Dr. A. B. Brown, New Orleans, La.; Secretary-Treasurer, Dr. George H. Matson, Columbus, Ohio; Executive Council, Dr. N. R. Coleman, Columbus, O.; Dr. James A. Duncan, Toledo, O.; Dr. Charles H. Cook, Natick, Mass.; Dr. Joseph G. Guernsey, Philadelphia, Pa.; Dr. W. Scott Nay, Underhill, Vt.

### TREASURY DEPARTMENT—BUREAU OF PUBLIC HEALTH AND MARINE-HOSPITAL SERVICE.

A board of commissioned medical officers will be convened at the Bureau of Public Health and Marine Hospital Service, 3 B street SE., Washington, D. C., Monday, May 22, 1911, at 10 o'clock a. m., for the purpose of examining candidates for admission to the grade of assistant surgeon in the Public Health and Marine-Hospital Service.

Candidates must be between 22 and 30 years of age, graduates of a reputable medical college, and must furnish testimonials from responsible persons as to their professional and moral character.

The following is the usual order of the examinations: 1, physical; 2, oral; 3, written; 4, clinical.

In addition to the physical examination, candidates are required to certify that they believe themselves free from any ailment which would disqualify them for service in any climate.

The examinations are chiefly in writing, and begin with a short autobiography of the candidate. The remainder of the written exercise consist in examination in the various branches of medicine, surgery and hygiene.

The oral examination includes subjects of preliminary education, history, literature, and natural sciences.

The clinical examination is conducted at a hospital, and when practicable, candidates are required to perform surgical operations on a cadaver.

Successful candidates will be numbered according to their attainments on examination, and will be commissioned in the same order as vacancies occur.

Upon appointment the young officers are ,as a rule, first assigned to duty at one of the large hospitals, as at Boston, New York, New Orleans, Chicago, or San Francisco.

After four years' service, assistant surgeons are entitled to examination for promotion to the grade of passed assistant surgeon.

Promotion to the grade of surgeon is made according to seniority and after due examination as vacancies occur in that grade.

Assistant surgeons receive $1,600, passed assistant surgeons $2,000, and surgeons, $2,500 a year. Officers are entitled to furnished quarters for themselves and their families, or, at stations where quarters cannot be provided, they receive commutation at the rate of thirty, forty, and fifty dollars a month, according to grade.

All grades above that of assistant surgeon receive longevity pay, 10 per cent. in addition to the regular salary for every five years' service up to 40 per cent. after twenty years' service.

The tenure of office is permanent. Officers traveling under orders are allowed actual expenses.

For further information, or for invitation to appear before the board of examiners, address, "Surgeon-General," Public Health and Marine-Hospital Servive, Washington, D. C."

---

### THE SECRET COMMISSION EVIL.

The giving and receiving of secret commissions has been discussed with increasing frequency for several years. Although the existence of this practice has long been recognized, we have always believed that it has been confined to a comparatively small number of physicians. Condemnation of this evil by the Association, by the Journal and by various medical societies, as well as occasional local investigations and exposures, have apparently failed to abolish it. Recent discussion shows that the better men in the profession appreciate the importance of the problem and the need of its solution by physicians. In this issue appear two articles on the subject, one the president's address before the Western Surgical Association, and the other an editorial from Colorado Medicine. Both of these articles are severe and carry the impression that the practice is widespread. We are loath to believe that conditions are as bad as represented. Yet this evil, if existent to any extent, should be freely discussed and unsparingly condemned. Its correction is of vital importance to the public as well as to physicians. While it has long been recognized as existent by the profession, the rapidly growing interest of the public in professional matters and the increasing disposition of physicians to take the people into their confidence, have appraised the public, apparently for the first time, of the existence of this condition. Now that the people are informed, the evil must be speedily corrected by the profession, for if it is not, the people themselves will demand the right to suppress it.

It is significant that, in spite of the specious arguments brought forward in defense of this practice in professional circles, no one has ever attempted to defend it before the public. The misleading excuses of "paying the physician or his time," "recognizing the diagnosis as of equal value with the operation," and other fallacious arguments which have been advanced in extenuation of this practice, will count for nothing with the public. Logically, they belong in the same category as the "poor boy" excuse for cheap medical colleges and night schools. Popularly, they will have much less weight, since the layman does not recognize, as yet, the importance of medical education to him and to his family, but does see, in the secret payment of commissions, that the patient, instead of being a sacred trust, is made an article of merchandise, whose money is divided without his knowledge.

The issue is so plain that it needs only to be stated to carry conviction to the unprofessional mind. It has been condemned and denounced repeatedly by medical organs and organizations and by high-minded medical men everywhere. Yet, we are told, the evil exists and is becoming more prevalent. If this be true, there is but one remedy, the remedy which in recent years has corrected many other pernicious and secret practices: Graft in the medical profession must receive the same treatment as graft in politics or in business—publicity. If the people and the profession are given the names of surgeons and specialists who offer secret commissions and of phyicians who accept them, this wretched business will soon come to an end.

Apparently there is one community in which this remedy is to be applied. The Denver Times, an evening paper, which has for a long time excluded all objectionable medical advertisements, says editorially in a recent issue:

"It is disquieting to learn from the organ of the Medical Society of Colorado that many physicians and surgeons are commercializing their practice through that system of secret rebates known as "fee-splitting." The method is about as follows: A patient consults a physician. The physician discovers that the case calls for the knowledge of a specialist or operation by a surgeon. Ethically it is the duty of the physician to seek the services of the most hihly qualified man. But the sight of some possible dollars blinds the vision to ethical considerations and the patient is sent to the specialist or surgeon who agrees to pay the pyhycian the largest percentage of his fee. It is a debasing custom of course; it admits of neither extenuation nor excuse; but it seems to be a custom to which numerous physicians and surgeons of repute are addicted, and we believe that it can only be abolished by the publication of the names of the men practicing it. Some of these names we have obtaned and authenticated already; we are investigating others now; and when the list is tolerably complete we will print it and gladly face the libel suits that such publication may involve. The men who do this kind of thing are well known already to the men who do not; in professional circles their names are a matter of common gossip; but they should be known equally well by the lay public, and we hope, shortly, to be able to give them the kind of advertising they merit.

And yet in this question, as in other complicated social problems, the public itself is to a certain extent to blame for existing conditions In the majority of communities, the family physician is today receiving the same compensation which was paid his professional forefathers two or three generations ago; and this in spite of the enormous increase of the cost of professional education and of the standard of living, as well as of the quality of medical services. Whether wisely or not, the physician fears in many cases to raise the price of his services or to charge on any other basis than the time-honored, but economically absurd, basis of the number of calls made on the patient. The result is that in many cases the patient pays willingly and promptly the large fee demanded by the surgeon, but settles his bill with his family physician grudgingly and after long delay. The family physician often gives far more in time and services to the patient than he can expect to receive compensation for; and he has consequently turned to the surgeon to receive as a gift what he should have received from the patient as a right. If the public will compensate the family physician fairly and promptly for his services and will insist that all transactions between the physician and the consultant be carried on with the full knowledge of the patient, the cause and the possibility of this evil will speedily disappear. Physicians and surgeons must recognize the existence of this evil, must admit the rights of the public in the matter and must lead the way in abolishing it, but the public, on its part, must recognize the conditions which have given rise to this problem, must do its share to help the profession to solve it, and must support the family physician, both financially and morally, so that he may be able to deal honestly and fairly with his patients.—*Jour. A. M. A., March* 11, 1911.

